# In Reply to “Letter Regarding ‘Granulomatous Inflammation and Hypercalcemia in Patients With Severe Systemic Oxalosis’”

**DOI:** 10.1016/j.ekir.2022.02.003

**Published:** 2022-02-11

**Authors:** Peggy Perrin, Jerome Olagne, Arnaud Delbello, Stanislas Bataille, Laurent Mesnard, Claire Borni, Bruno Moulin, Sophie Caillard

**Affiliations:** 1 Department of Nephrology and Transplantation, University Hospital, Strasbourg, France; 2 Fédération de Médecine Translationnelle (FMTS), Strasbourg, France; 3 Institut National de la Santé et de la Recherche Médicale (INSERM) U1109, LabEx TRANSPLANTEX, Strasbourg, France; 4 Department of Pathology, University Hospital, Strasbourg, France; 5 Département de Néphrologie, Dialyse et Transplantation d’Organes, Centre Hospitalier et Universitaire de Toulouse, Toulouse, France; 6 Institut National de la Santé et de la Recherche Médicale—Centre de Physiopathologie Toulouse Purpan, Institut National de la Santé et de la Recherche Médicale (INSERM) UMR 1043—Centre National de le Recherche Scientifique (CNRS) 5282, Toulouse, France; 7 Université Paul Sabatier Toulouse III, Toulouse, France; 8 Phocean Institute of Nephrology, Marseille, France; 9 ELSAN, Clinique Bouchard, Marseille, France; 10 Aix-Marseille Univ, C2VN, Institut National de la Santé et de la Recherche Médicale (INSERM), INRAE, Marseille, France; 11 Service des Soins Intensifs Néphrologiques et Rein Aigu, Department of Nephrology and Transplantation, Hôpital Tenon, APHP Sorbonne Université, Paris, France; 12 AURAL 15, Place du Capitaine DREYFUS, Colmar, France

The Authors Reply:

We recently reported a case series of 5 patients with primary hyperoxaluria and emphasized the importance of granulomatous inflammation in severe systemic oxalosis.^1^ All cases presented diffuse hypermetabolic lesions on fluorodeoxyglucose-positron emission tomography/computed tomography and hypercalcemia. Hypermetabolic foci corresponded to areas of granulomatous inflammation elicited by calcium oxalate crystals. We thank Halfon *et al.* for their interest in our study. In line with the results from us and other groups,^2^ they describe a patient with primary hyperoxaluria, hypercalcemia, and high 1,25 (OH)_2_ vitamin D levels who was successfully treated with corticosteroids.^1^ In our study, there was no report of vitamin C treatment. Some aspects of the clinical management of this condition require further discussion. During follow-up, we occasionally detected severe hypercalcemia in 3 of the 5 study patients (cases numbers 1, 2, and 3); notably, hypercalcemia in case number 3 resulted in life-threatening coma. High doses of steroids given for induction immunosuppression or acute graft rejection were only temporarily successful in controlling hypercalcemia (cases numbers 1, 2, and 3). Unfortunately, hypercalcemia recurred when corticosteroids were tapered under 15 mg/kg/d. Furthermore, a maintenance therapy with 7.5 mg/d of steroids in cases number 1 and 2 did not prevent recurrence.

Considering the elevation of bone resorption markers, the presence of lytic bone lesions, and the high incidence of fractures observed in our study, bone antiresorptive agents (e.g., bisphosphonates or denosumab that is not renally cleared) may be a part of the therapeutic armamentarium to control hypercalcemia and to prevent fractures in patients with systemic oxalosis.
